# Cognitive Enhancer Effects of Low Memantine Doses Are Facilitated by an Alpha7 Nicotinic Acetylcholine Receptor Agonist in Scopolamine-Induced Amnesia in Rats

**DOI:** 10.3389/fphar.2019.00073

**Published:** 2019-02-05

**Authors:** Zsolt Kristóf Bali, Nóra Bruszt, Sai Ambika Tadepalli, Roland Csurgyók, Lili Veronika Nagy, Márton Tompa, István Hernádi

**Affiliations:** ^1^Department of Experimental Zoology and Neurobiology, Faculty of Sciences, University of Pécs, Pécs, Hungary; ^2^János Szentágothai Research Center, Center for Neuroscience, University of Pécs, Pécs, Hungary; ^3^Institute of Physiology, Medical School, University of Pécs, Pécs, Hungary

**Keywords:** combination drug therapy, alpha7 nicotinic acetylcholine receptor, memantine, spatial memory, scopolamine, behavior

## Abstract

Alpha7 nicotinic acetylcholine receptors (nAChRs) play an important role in learning and memory and are promising targets for pharmacological cognitive enhancement. Memantine, an approved substance for Alzheimer’s disease treatment, is an antagonist of the N-Methyl-D-aspartate receptor (NMDAR) and also acts as an alpha7 nAChR antagonist. Here, we tested the interaction between an alpha7 nAChR agonist (PHA-543613) and memantine. Efficacy of memantine, PHA-543613, and their co-administration were investigated on the spatial working memory of rats using the spontaneous alternation paradigm in T-maze. Scopolamine-induced transient amnesia was used to model cognitive impairment. First, the dose-response relationship was assessed for memantine, and its lowest effective dose was found to be 0.1 mg/kg. Then, co-administration treatments with subeffective doses of the alpha7 nAChR agonist PHA-543613 and different doses of memantine were tested. The co-administration of subeffective drug doses significantly improved memory performance of the rats and reversed scopolamine-induced deficits. Interestingly, a higher than effective (0.3 mg/kg) dose of memantine did not increase performance in monotreatment, only in co-administration with PHA-543613. However, the co-administration of PHA-543613 did not further increase the efficacy of the previously effective monotreatment doses of memantine. Thus, the efficacy of memantine monotreatment and its co-administration with PHA-543613 converged to create a common ceiling effect, with an additive interaction found in the behavioral effects. These results suggest that memantine and PHA-543613 may exert their cognitive enhancer effects on the same target, possibly on the alpha7 nAChRs. Results also suggest possible benefits of a combination therapy with memantine and alpha7 nAChR agonists.

## Introduction

Globally, 24.3 million people suffer from dementia, with approximately 4.6 million new cases every year (Ferri et al., 2005). This has led to an increased global need in studying dementia, or cognitive impairment, and its underlying mechanisms. A major area of concern is development of effective pharmacological treatments that produce minimal or no adverse side effects.

Memantine, an uncompetitive antagonist of the N-methyl-D-aspartate receptor (NMDAR), is an approved drug in the treatment of Alzheimer’s disease (AD). Memantine has also been found effective in enhancing memory performance in various animal models of dementia and different behavioral paradigms (Wise and Lichtman, 2007; Busquet et al., 2012; Schneider et al., 2013). Memantine is thought to block over-stimulation of the NMDARs caused by amyloid-beta (Aβ) oligomers implicated in the pathogenesis of AD (Danysz and Parsons, 2012) without affecting physiological glutamatergic activity necessary for synaptic plasticity (Parsons et al., 1999). However, it was also observed to act on alpha7 nicotinic acetylcholine receptors (nAChRs) as an antagonist, perhaps more potently than on NMDARs (Aracava et al., 2005).

Selective alpha7 nicotinic acetylcholine receptor (nAChR) agonists are another class of drugs that are emerging as potent treatments for neurocognitive disorders. A substantial body of evidence indicates the role of alpha7 nAChRs in cognitive performance and memory (Dani and Bertrand, 2007). Thus, they provide a suitable target for the treatment of AD and other neurocognitive disorders (Toyohara and Hashimoto, 2010). PHA-543613 is a selective alpha7 nAChR agonist, which reportedly alleviates amphetamine-induced auditory gating deficit and improves object recognition memory in rats (Wishka et al., 2006). It also appears to be effective in treating Aβ_25-35_-mediated cognitive deficits in mice (Sadigh-Eteghad et al., 2015). In addition, it ameliorates spatial working memory deficits induced by the muscarinic AChR antagonist scopolamine in rats (Bali et al., 2015).

While most of the therapeutic treatments available today involve a single cognitive enhancer agent, there is an increasing interest in developing combined treatments that involve two or more cognitive enhancers in otherwise subeffective or inactive doses. A fixed-dose combination of memantine and acetylcholinesterase inhibitor (AChEI) donepezil has already been approved by the Food and Drug Administration (FDA) of the United States. However, the same therapy has not been approved by the European Medicines Agency because of limited clinical evidence of its benefits over monotherapies with AChEIs or memantine (Deardorff and Grossberg, 2016). Combinations of memantine and donepezil also showed limited efficacy in preclinical animal models (Wise and Lichtman, 2007; Woodruff-Pak et al., 2007). However, galantamine, another AChEI also possessing affinity for an allosteric site of the alpha7 nAChR, exerted far better synergistic effects in co-administration with memantine in alleviating scopolamine-induced cognitive deficits in mice (Busquet et al., 2012). It has also been reported that alpha7 nAChR activity enhances glutamatergic signaling via NMDARs (Yang et al., 2013; Bali et al., 2017).

Based on the above findings, we hypothesized that various pharmacological interactions might occur between memantine and alpha7 nAChR agonists. Such interactions could also potentially result in an increased efficacy of combinational treatments on cognitive performance. Therefore, in the present study, our aim was to test the efficacy of co-administration treatments with the selective alpha7 nAChR agonist PHA-543613 and memantine on spatial working memory performance in the scopolamine-induced transient amnesia model in rats.

## Materials and Methods

### Animals

Twenty-four 7- to 12-month old male Long Evans (LE) rats (weighing 380–520 g) and eleven 4- to 7-month old male Wistar (W) rats (weighing 350–450 g) were used in the present study. Animals were obtained from Charles River Laboratories and were housed in pairs in individually ventilated cages under controlled conditions in the animal house of the Szentágothai Research Centre, University of Pécs (12/12 h light/dark cycle, with controlled temperature and humidity). Rats were fed daily with 17 g of laboratory chow per animal per day (on experimental days only after they had been tested) throughout the experiments to ensure sufficient motivation for exploration in the testing apparatus. Water was available ad libitum. This study was carried out in accordance with the recommendations of Decree No. 40/2013. (II. 14.) of the Hungarian Government and EU Directive 2010/63/EU on the protection of animals used for scientific purposes. The protocol was approved by the Animal Welfare Committee of the University of Pécs (Licence No. BA02/2000-25/2015).

### T-maze Apparatus and Spontaneous Alternation Test Procedure

The T-maze apparatus was constructed according to Deacon and Rawlins (2006), and the dimensions were described in our previous study (Bali et al., 2015). The applied experimental protocol was based on the study of Spowart-Manning and van der Staay (2004) with some modifications made in our laboratory (Bali et al., 2015). The rat was placed in the start arm and after the opening of the guillotine door, he had to make a choice between the right and left goal arms. After exploring the goal arm and returning to the start arm, the rat was confined in the start arm for 10 s between two trials. Then, the trial was repeated. Each session lasted for a maximum of 25 min or a maximum of 15 consecutive trials. A session was considered invalid if fewer than nine trials were completed. Because of the within-subject statistical design, animals that had any invalid sessions, were excluded from the analysis of the entire experiment.

In each trial, we primarily recorded which goal arm was chosen by the rat. If the rat entered the opposite arm compared to the previous trial, the choice was considered a correct choice (alternation). Otherwise, it was considered an erroneous choice. Alternation rate was determined as the proportion of correct arm choices (alternations) and the total number of trials offered for alternation (a maximum of 14 if all 15 trials were run).

In addition, as secondary endpoints, average trial duration (time elapsed from door opening until returning to the starting position) and average choice latency (time elapsed from door opening until entering one of the goal arms) were determined to control for side effects of the pharmacological treatments not related to memory.

### Drugs and Routes of Administration

Scopolamine hydrobromide (Tocris), PHA-543613 hydrochloride (Tocris), and memantine hydrochloride (Tocris) were dissolved in physiological saline to create a final injection volume of 1 ml/kg. Scopolamine was injected intraperitoneally (i.p.) 10 min before experimental sessions, and PHA-543613 and memantine were injected subcutaneously (s.c.) 40 min before experiments (30 min before scopolamine administration). In treatments, where scopolamine, memantine and/or PHA-543613 were not administered, compounds were replaced with saline (vehicle, VEH) injected through the corresponding route of administration. In co-administration treatments, memantine and PHA-543613 were administered consecutively in two separate subcutaneous injections performed on the opposite sides of the body.

### Experimental Design

Experiments were designed to investigate possible pharmacological interactions between memantine and the alpha7 nAChR agonist PHA-543613 in their effect on spatial working memory. Therefore, subeffective, effective, and higher than effective doses of memantine were co-administered with subeffective doses of PHA-543613. The efficacy of the co-administration treatments was compared with that of monotreatments with memantine and PHA-543613. Efficacy of cognitive enhancer treatments were tested against transient amnesia induced by scopolamine (0.5 mg/kg).

The study consisted of two phases: (1) an experiment for the determination of subeffective and effective doses of memantine; and (2) a set of experiments testing the interactive effects of memantine and PHA-543613 at different doses.

The doses applied in the memantine efficacy test were chosen on the basis of preceding pilot experiments and were the following: 0.001, 0.003, 0.01, 0.03, and 0.1 mg/kg. Subeffective doses of PHA-543613 were determined according to our previous study (Bali et al., 2015). Each experiment was preceded by 3–6 sessions for habituation and training without pharmacological treatment until rats achieved stable control performance in the spontaneous alternation task.

In the experiments testing co-administration of memantine and PHA-543613 (*Experiments 1*–*4*, Table 1), the following pharmacological treatments were applied: scopolamine alone (further referred to as Scop), memantine monotreatment in different doses followed by scopolamine (Mem[dose]), PHA monotreatment in subeffective doses followed by scopolamine (PHA[dose]), and co-administration treatment with memantine and PHA followed by scopolamine (Mem[dose]+PHA[dose]). All animals in a given experiment were subjected to each treatment. The treatments were applied in a counterbalanced (Latin square) design to achieve a fully randomized sequence of different treatments. The rat strains and doses applied in the experiments are listed in Table 1.

**Table 1:** Summary of the rat strains and the doses of pharmacological compounds used in experiments performed for the evaluation of combined treatments with memantine and PHA-543613.

	Strain	Vehicle	Memantine (mg/kg)	PHA-543613 (mg/kg)	Scopolamine (mg/kg)
Experiment 1	Long Evans	Saline	0.003	0.1	0.5
Experiment 2	Long Evans	Saline	0.03	0.1	0.5
Experiment 3	Wistar	Saline	0.1	0.3	0.5
Experiment 4	Wistar	Saline	0.3	0.3	0.5


### Data Analysis and Statistics

The control performance of every animal was determined as its average alternation rate during training sessions. In the analysis of experiments with pharmacological treatments, data of animals that performed above the predetermined 0.6 alternation rate after Scop treatment (i.e., showing no considerable memory impairment), were excluded from all treatments. Thus, the effects of memantine, PHA-543613 and their co-administration were only tested on animals with confirmed sensitivity to scopolamine. Statistical analyses were performed using the IBM SPSS 20.0 statistical program and MS Excel. Alternation rate, average trial duration, and average choice latency data were analyzed with repeated measures ANOVA. Following significant main effects of treatments, different treatments (Control, Mem, PHA, Mem+PHA) were compared with the scopolamine treatment using *post hoc* LSD test. Then, the *p*-values were corrected with Holm’s method for multiple comparisons (Holm, 1979). Alternation rate after a given treatment was also compared to the chance level (0.5) using one-sample *t*-test with a one-tailed null hypothesis. An alternation rate significantly higher than the chance level indicated that rats showed normal alternating behavior and good memory performance after a given treatment. Effect size of treatments was estimated using eta squared (η^2^) in ANOVA models, and Cohen’s d for correlated samples (d_rm_) in pairwise comparisons as calculated according to Lakens (2013).

Since initial data showed a marked difference between animals in their responsiveness to memantine (i.e., optimal dose varied), which also affected the effectiveness of co-administration treatments, we performed an additional pooled analysis of cases when memantine was not effective in monotreatment. Thus, subjects from all the experiments with an alternation performance of less than 0.6 after memantine monotreatment were included in the pooled analysis. With the pooled data, we also performed an analysis to test whether memantine and PHA-543613 interacted in an additive or superadditive (synergistic) manner when used in co-administration (Bali et al., 2017). We calculated the sum of the memory enhancer effect of memantine and PHA-543613 monotreatments in comparison to the scopolamine alone treatment ([Mem]-[Scop] and [PHA]-[Scop], respectively) and compared it to the effect of their co-administration ([Mem+PHA]-[Scop]) using the paired samples *t*-test. In all statistical analyses, the level of significance was *p* < 0.05. All data used in the statistical analysis are available on Mendeley Data (Bali et al., 2018).

## Results

### Dose–Effect Relationship of Memantine

**FIGURE 1:**
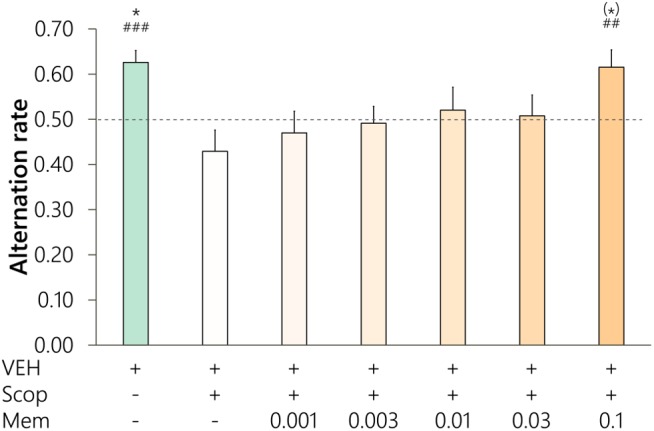
Dose-response relationship for memantine against scopolamine-induced (0.5 mg/kg, i.p.) amnesia in the spontaneous alternation task (*n* = 9, Long Evans rats). Text below the graph represents the injections given before testing a given treatment, also showing the dose of memantine (mg/kg, s.c.). Significance level of *post hoc* comparisons between a given treatment and scopolamine alone treatment were marked with asterisks above the bars: (^∗^) *p* < 0.1, ^∗^*p* < 0.05. Hash symbols mark that the alternation performance after the given treatment was significantly higher than the chance level (0.5, dashed line): ^##^*p* < 0.01, ^###^*p* < 0.001.

Experiments were performed to determine the relationship between the dose of memantine and its cognitive enhancer effect against scopolamine (Figure 1). Out of the initial 13 LE rats, two animals were excluded from the statistical analysis because of invalid sessions, and another two were excluded because of the lack of effects of Scop on memory performance. A significant main effect of pharmacological treatments was found on the alternation rate of rats in the T-maze [*n* = 9, *F*(3.2, 25.8) = 3.12, *p* = 0.040, η^2^ = 0.239]. Control performance of rats was above the chance level (one-sample *t* = 4.745, *p* < 0.001) and was significantly higher than after scopolamine treatment [Control vs. Scop: 0.63 ± 0.03 (mean ± SEM) vs. 0.43 ± 0.05, *p* = 0.047, d_rm_ = 1.721] indicating that rats showed good control memory performance and alternating behavior. Memantine dose-dependently attenuated scopolamine-induced memory impairment and increased the average alternation rate of rats. Although the memory enhancing effect of memantine in the dose of 0.1 mg/kg was only marginally significant compared with the scopolamine alone treatment according to the corrected *p*-value (Mem0.1 vs. Scop: 0.62 ± 0.04 vs. 0.43 ± 0.05, *p* = 0.073, d_rm_ = 1.444), Mem0.1 treatment restored normal alternating behavior of animals (one-sample *t* = 3.011, *p* = 0.008). Therefore, 0.1 mg/kg dose of memantine was considered as an effective dose for cognitive enhancement.

### Experiment 1: Memantine and PHA-543613 in Subeffective Doses

**FIGURE 2:**
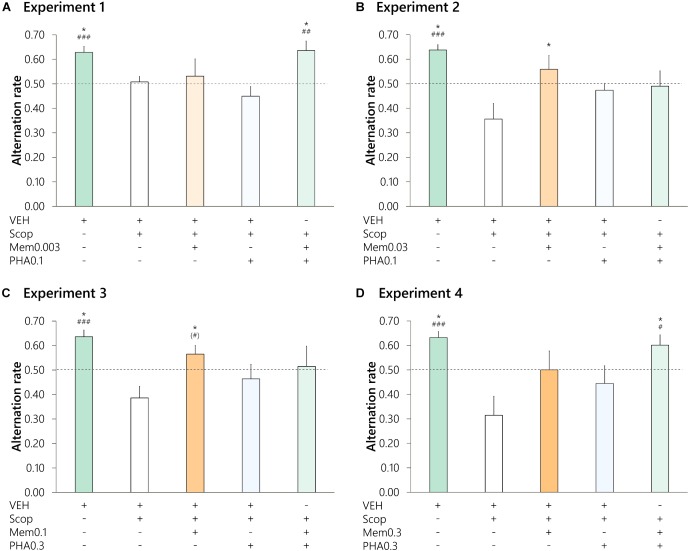
Effects of different doses of memantine and PHA-543613 in monotreatments and in different combinations against scopolamine-induced (0.5 mg/kg, i.p.) amnesia. Text below the graph represents the injections given before testing a given treatment. Dose of memantine (s.c.) and PHA-543613 (s.c.) in the given experiment is shown after their abbreviation (Mem, PHA, respectively) in mg/kg. **(A)** In *Experiment 1*, a subeffective dose of memantine was combined with a subeffective dose of PHA-543613 (*n* = 9 Long Evans rats); **(B,C)** In *Experiments 2* and *3*, effective doses of memantine were combined with subeffective doses of PHA-543613 (*n* = 10 Long Evans rats and 8 Wistar rats, respectively); **(D)** In *Experiment 4*, a subeffective dose of PHA-543613 was tested with a dose of memantine that was higher than its most effective dose (*n* = 8 Wistar rats). Significance level of *post hoc* comparisons between a given treatment and scopolamine alone treatment were marked with asterisks above the bars: ^∗^*p* < 0.05. Hash symbols mark the significance level of the difference between the alternation performance after the given treatment and the chance level (0.5, dashed line): (#) *p* < 0.1, ^#^*p* < 0.05, ^##^*p* < 0.01, ^###^*p* < 0.001.

In *Experiment 1* (Figure 2A), the subeffective 0.003 mg/kg dose of memantine was tested in co-administration with the subeffective 0.1 mg/kg dose of PHA-543613 against scopolamine-induced amnesia of rats. Experiments were performed on altogether 12 LE rats. One animal was excluded because of invalid sessions, and another two animals were excluded because of the lack of memory impairment after Scop treatment. Following the significant main effect of the pharmacological treatments [*n* = 9, *F*(4, 32) = 3.910, *p* = 0.011, η^2^ = 0.266], the significant difference between the alternation rate after VEH and Scop treatments validated the model for cognitive impairment (Control vs. Scop: 0.63 ± 0.02 vs. 0.51 ± 0.02, *p* = 0.041, d_rm_ = 1.723). Monotreatments with memantine or PHA-543613 were not effective enough to attenuate the scopolamine-induced memory deficit (0.53 ± 0.07 and 0.45 ± 0.04, respectively; Mem0.003 vs. Scop: *p* > 0.1, d_rm_ = 0.162; PHA0.1 vs. Scop: *p* > 0.1, d_rm_ = -0.617), and alternation performance did not significantly exceed the chance level neither after Mem0.003 nor after PHA0.1 treatment.

However, the co-administration of these subeffective doses of memantine and PHA-543613 resulted in a significant increase in the alternation rate (Mem0.003+PHA0.1: 0.64 ± 0.04; Mem0.003+PHA0.1 vs. Scop: *p* = 0.043, d_rm_ = 1.338), and it restored the normal memory performance of the animals (one-sample *t* = 3.506, *p* = 0.004). Thus, in *Experiment 1* an interaction between subeffective doses of memantine and PHA-543613 was found to be beneficial for the enhancement of cognitive performance of LE rats.

### Experiment 2: An Effective Dose of Memantine and a Subeffective Dose of PHA-543613

In *Experiment 2* (Figure 2B), the subeffective dose of PHA-543613 (0.1 mg/kg) was co-administered with 0.03 mg/kg memantine, a dose that was considered initially as subeffective according to the dose–response curve of memantine (see section Dose–effect Relationship of Memantine). Two of the initial 12 animals were excluded because of the lack of memory impairment after Scop. Surprisingly, in *Experiment 2*, scopolamine-induced memory impairment of LE rats [*n* = 10; *F*(4, 36) = 5.190, *p* = 0.002, η^2^ = 0.284; Control vs. Scop: 0.64 ± 0.02 vs. 0.36 ± 0.06, *p* = 0.006, d_rm_ = 1.778] was attenuated by Mem0.03 treatment (Mem0.03: 0.56 ± 0.06; Mem0.03 vs. Scop: p = 0.035, d_rm_ = 1.070). However, this enhancement of the memory performance was not sufficient to increase the alternation rate above the chance level and to restore normal alternating behavior (one-sample *t* = 1.057, *p* > 0.1). The co-administration of Mem0.03 and PHA0.1 treatments was not at all effective against the scopolamine-induced deficit, as the alternation performance after the co-administration treatment was not significantly higher than the scopolamine alone treatment, and the alternation rate was close to the chance level (Mem0.03+PHA0.1: 0.49 ± 0.06; Mem0.03+PHA0.1 vs. Scop: *p* > 0.1, d_rm_ = 0.671). Thus, the addition of PHA-543613 to an effective dose of memantine did not improve but rather attenuated its memory enhancing effect.

### Experiment 3: An Effective Dose of Memantine and a Subeffective Dose of PHA-543613

In *Experiment 3* (Figure 2C), we investigated the effects of 0.1 mg/kg memantine alone and in co-administration with PHA-543613 (0.3 mg/kg) in W rats. Earlier, the dose of memantine was found to be an effective dose in LE rats. Twelve rats were tested in the experiments, and two animals were excluded because of invalid sessions, while another two were excluded because of the lack of memory impairment after Scop. Pharmacological treatments produced a significant main effect [*n* = 8, *F*(4, 28) = 2.957, *p* = 0.037, η^2^ = 0.262], and scopolamine induced a decrease of the performance sufficient for modeling cognitive impairment (Control vs. Scop: 0.64 ± 0.03 vs. 0.39 ± 0.05, *p* = 0.026, d_rm_ = 2.300). As in LE rats, Mem0.1 treatment increased the alternation rate of W rats compared with the performance observed after scopolamine alone treatment (Mem0.1: 0.56 ± 0.03; Mem0.1 vs. Scop: *p* = 0.048, d_rm_ = 1.505). Moreover, Mem0.1 treatment restored normal memory performance, resulting in an alternation rate marginally significantly higher than the chance level (one-sample t = 1.859, *p* = 0.053). The 0.3 mg/kg dose of PHA-543613 was not effective against scopolamine-induced memory impairment (PHA0.3: 0.46 ± 0.06, PHA0.3 vs. Scop: *p* > 0.1, d_rm_ = 0.513). A co-administration treatment with an effective dose of memantine and a subeffective dose of PHA-543613 (Mem0.1+PHA0.3: 0.52 ± 0.08) only slightly attenuated the scopolamine-induced deficit on average. The effect was not significant compared with that of the scopolamine alone treatment (Mem0.1+PHA0.3 vs. Scop: *p* > 0.1, d_rm_ = 0.696), and it did not restore normal alternation behavior (one-sample *t* = 0.182, *p* > 0.1). Again, satisfactory efficacy of memantine could not be further potentiated with the addition of PHA-543613.

### Experiment 4: Memantine in a Higher Than Effective Dose

In *Experiment 4* (Figure 2D), the behavioral pharmacology of memantine was investigated in W rats at a dose of 0.3 mg/kg, which was not tested in monotreatment earlier but was higher than the lowest effective dose in LE rats. The dose of PHA-543613 was also 0.3 mg/kg, which was similar to *Experiment 3*. Three of the initial 11 animals were excluded because of invalid sessions, and all rats showed memory impairment after Scop. Treatments in *Experiment 4* resulted in a significant main effect [*n* = 8, *F*(4, 28) = 3.693, *p* = 0.015, η^2^ = 0.322], and scopolamine effectively impaired the performance of rats (Control vs. Scop: 0.63 ± 0.02 vs. 0.31 ± 0.08, *p* = 0.024, d_rm_ = 1.958). Although memantine treatment slightly improved the alternation rate on average, the resulting performance (Mem0.3: 0.50 ± 0.08) was not significantly higher either compared with scopolamine treatment (Mem0.3 vs. Scop: *p* > 0.1, d_rm_ = 0.848) or to the chance level (one-sample t = 0.002, *p* > 0.1). As in *Experiment 3*, 0.3 mg/kg dose of PHA-543613 was found to be subeffective for the memory impairment induced by scopolamine in W rats (PHA0.3: 0.44 ± 0.07; PHA0.3 vs. Scop: *p* > 0.1, d_rm_ = 0.613). However, the co-administration of the ineffective doses of memantine and PHA-543613 resulted in a significant improvement of alternation performance of rats compared with scopolamine alone treatment (Mem0.3+PHA0.3: 0.60 ± 0.04; Mem0.3+PHA0.3 vs. Scop: *p* = 0.040, d_rm_ = 1.611). The co-administration treatment also restored normal memory performance and resulted in an average alternation rate significantly higher than the chance level (one-sample *t* = 2.391, *p* = 0.024). Thus, PHA-543613 positively influenced the efficacy of memantine also at higher than effective doses of memantine.

### Comparison of Effect Sizes as a Function of the Dose of Memantine

**FIGURE 3:**
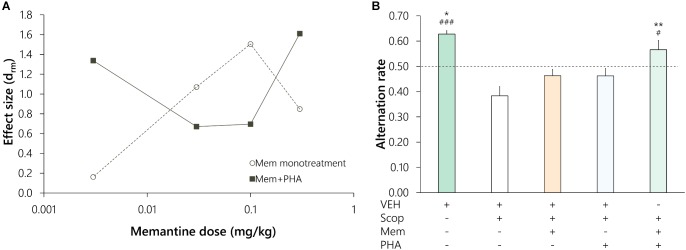
Comparison of effect sizes of memantine (Mem) monotreatments and its combinations with PHA-543613 (PHA) against scopolamine-induced amnesia **(A)**, and pooled analysis of cases (*n* = 26) with non-effective memantine monotreatments **(B)**. On part **(B)**, significance level of *post hoc* comparisons between a given treatment and scopolamine alone treatment were marked with asterisks above the bars: ^∗^*p* < 0.05, ^∗∗^*p* < 0.01. Hash symbols mark that the alternation performance after a given treatment was significantly higher than the chance level (0.5, dashed line): ^#^*p* < 0.05, ^###^*p* < 0.001.

For the purpose of standardized comparison of efficacy of treatments in different experiments, we compared effect sizes for memantine (Mem vs. Scop) in monotreatments and in co-administration with PHA-543613 (Mem+PHA vs. Scop) as a function of the memantine dose (Figure 3A). The effect size of memantine monotreatment showed an inverted U-shaped relation with the dose, as the effect size increased in the 0.003 mg/kg to 0.1 mg/kg dose range, while a higher dose (0.3 mg/kg) resulted in the decrease of the effect size. Effect sizes of co-administration treatments (Mem+PHA) changed conversely as a result of increasing memantine doses. Mem+PHA treatment exerted a substantial cognitive enhancing effect when memantine was applied in a lower (0.003 mg/kg) or in a higher (0.3 mg/kg) dose, while at intermediate memantine doses, co-administration treatment exerted a smaller effect (from 0.03 to 0.1 mg/kg).

### Pooled Analysis of Cases With Ineffective Memantine Doses

To test the hypothesis that PHA-543613 potentiates ineffective doses of memantine, we performed a pooled analysis of data from animals that were not positively affected by the applied dose of memantine monotreatment. Results (Figure 3B) showed significant main effect of the treatments [*n* = 26; *F*(3.03, 75.8) = 10.774, *p* < 0.001, η^2^ = 0.301]. Scopolamine-induced decrease of the alternation performance (Control vs. Scop: 0.63 ± 0.01 vs. 0.38 ± 0.04, *p* < 0.001, d_rm_ = 1.781) was not significantly attenuated by either memantine or PHA-543613 monotreatment (Mem: 0.46 ± 0.02, Mem vs. Scop: *p* > 0.1, d_rm_ = 0.501; PHA: 0.46 ± 0.03, PHA vs. Scop: *p* > 0.1, d_rm_ = 0.468). However, co-administration treatment with memantine and PHA-543613 significantly enhanced memory performance of the animals compared with the scopolamine alone treatment (Mem+PHA: 0.57 ± 0.04, Mem+PHA vs. Scop: *p* = 0.003, d_rm_ = 0.969), and significantly increased the alternation rate above the chance level (*t* = 1.773, *p* = 0.044).

Furthermore, we assessed the nature of the interaction between behavioral effects of memantine and PHA-543613. We found that the effect of memantine and PHA-543613 in co-administration (0.18 ± 0.05) was not significantly higher than the sum of monotreatment effects (0.16 ± 0.09; *t* = 0.324, *p* = 0.748). Hence, pooled analysis revealed that the co-administration of memantine and PHA-543613 provide a beneficial additive effect when memantine is used at an ineffective dose.

### Lack of Effects on Time Variables

No significant main effect of pharmacological treatments was found on the measured time variables in most experiments, neither in the experiment determining effective doses of memantine [choice latency: *F*(2.2, 17.3) = 2.620, *p* = 0.098; trial time: *F*(6, 48) = 0.367, *p* = 0.897], nor in most experiments with co-administration treatments (data are summarized in Tables 2, 3). One exception was *Experiment 4*, where a significant main effect [*F*(4, 28) = 2.733, *p* = 0.049] of pharmacological treatments was found on the average choice latency of the animals. However, in *post hoc* comparisons of time variables, neither monotreatments nor the co-administration treatment were significantly different from control sessions (29.4 ± 3.6, n.s.) or the scopolamine alone treatment (25.7 ± 9.6, n.s.). We can conclude that the applied pharmacological treatments did not generally affect the speed of animals in performing the task.

**Table 2:** Effects of scopolamine (Scop, 0.5 mg/kg, i.p.) and memantine (Mem followed by the dose in mg/kg, s.c.) on the average choice latency and the average trial time data (mean ± SEM, *n* = 9).

	Control	Scop	Mem0.001	Mem0.003	Mem0.01	Mem0.03	Mem0.1
Average choice latency	27.1 s	18.2 s	27.0 s	7.7 s	15.7 s	11.7 s	16.8 s
	±2.4 s	±5.4 s	±8.8 s	±2.0 s	±4.6 s	±2.0 s	±4.0 s
Average trial time	71.1 s	58.4 s	75.6 s	61.3 s	61.4 s	67.0 s	66.2 s
	±5.3 s	±13.1 s	±14.6 s	±11.0 s	±8.3 s	±11.3 s	±9.0 s


**Table 3:** Means and standard errors of average choice latency and average trial time data after different treatments in Experiments 1–4.

		Control	Scop	Mem	PHA	Mem+PHA
Average choice latency	Experiment 1	32.0 s	21.6 s	42.7 s	22.6 s	30.4 s
		±3.9 s	±6.6 s	±14.1 s	±6.4 s	±9.9 s
	Experiment 2	34.7 s	17.9 s	24.2 s	19.0 s	23.5 s
		±3.7 s	±6.1 s	±7.5 s	±5.5 s	±6.7 s
	Experiment 3	17.0 s	14.0 s	10.9 s	22.8 s	24.8 s
		±2.0 s	±3.1 s	±1.6 s	±9.1 s	±10.3 s
	Experiment 4	29.4 s	25.7 s	19.7 s	39.7 s	21.3 s
		±3.6 s	±9.6 s	± 4.9 s	±4.9 s	±5.2 s
Average trial time	Experiment 1	73.6 s	58.2 s	77.2 s	51.7 s	69.8 s
		±5.1 s	±6.3 s	±16.8 s	±11.5 s	±11.2 s
	Experiment 2	79.3 s	66.5 s	64.5 s	65.2 s	76.5 s
		±6.8 s	±12.3 s	±13.6 s	±9.2 s	±11.5 s
	Experiment 3	94.6 s	81.9 s	64.9 s	89.1 s	87.3 s
		±8.1 s	±9.4 s	±9.2 s	±15.6 s	±11.6 s
	Experiment 4	71.1 s	79.0 s	68.1 s	85.3 s	73.2 s
		±6.4 s	±6.9 s	±9.6 s	±9.2 s	±12.5 s


*Sample sizes in Experiments 1–4 were 9, 10, 8, and 8, respectively.*

## Discussion

The present study investigated interactions between memantine and the alpha7 nAChR agonist PHA-543613 in their cognitive effects on rats testing the hypothesis that a combinational treatment would lead to increased efficacy in ameliorating cognitive deficits. The dose–effect relationship of low memantine doses was assessed, and the maximum efficacy of memantine was found at the 0.1 mg/kg dose, while higher doses (in *Experiment 4*) exerted no memory enhancement. Such an inverted U-shaped dose–response relationship of pharmacological cognitive enhancement is typical (Harada et al., 2012; Gould et al., 2013) as it has also been reported previously with memantine (Woodruff-Pak et al., 2007; Barber et al., 2010; Schneider et al., 2013). Although much higher doses of memantine were frequently used in rodent behavioral experiments (up to 30 mg/kg), 5 mg/kg is the maximum therapeutically relevant acute dose of memantine in rats, because it results in plasma levels that correspond to the well-tolerated upper limit in human patients (Parsons et al., 2007; Rammes et al., 2008). Conversely, high memantine doses supposedly result in non-selective receptor binding and induce side-effects similarly to other NMDAR antagonists like MK-801 (Parsons et al., 1999). In fact, such non-selective physiological changes and adverse effects on behavior were reported already at higher than 1 mg/kg memantine doses (Wise and Lichtman, 2007; Ihalainen et al., 2011; Kotermanski et al., 2013; Schneider et al., 2013). Similar to the present findings, earlier results also support the effectiveness of memantine in low doses below 1.0 mg/kg (Wise and Lichtman, 2007). Thus, using low doses of memantine in our experiments was relevant for the purpose of testing the effects of memantine in co-administration treatments and non-specific (e.g., increase of ACh level) and adverse effects were presumably also avoided. According to the results in the secondary measurements (choice latency, trial time), no adverse effects of the memantine treatments were found, indicating that memantine did not influence the speed of animals while performing the task.

According to the established memantine dose–effect curve, we tested co-administration treatments of effective and ineffective doses of memantine with subeffective doses of alpha7 nAChR agonist PHA-543613. Results showed that the co-administration treatment produced a more beneficial effect than monotreatments when memantine was applied in ineffective doses, regardless of whether the dose was lower (0.003 mg/kg) or higher (0.3 mg/kg) than the effective dose. Pooled analysis of ineffective memantine monotreatments revealed that the cognitive enhancer effects of memantine and PHA-543613 are additive when the agents are used in co-administration. However, PHA-543613 failed to further potentiate effective doses of memantine (0.03 and 0.1 mg/kg), as these co-administration treatments did not significantly increase memory performance compared with scopolamine alone treatments. These results might raise a possible interpretation that an antagonistic relationship was found between memantine and PHA-543613 with effective memantine monotreatments, although the performance after the co-administration treatment was not significantly worse than that after the monotreatment with memantine in the same experiment. Effect size comparisons of memantine monotreatments and co-administration treatments in all four experiments implied that the maximum effect of memantine monotreatment and its co-administration with PHA-543613 converged to a common ceiling effect. Thus, the addition of an alpha7 nAChR agonist might help to reach maximum efficacy of memantine in a wider dose range. However, it cannot increase the efficacy of memantine beyond that of an optimal monotreatment dose. Note that the limitation of such interpretations is that the animals used in the present study were not inherently impaired in cognition, and the scopolamine-induced amnesia model mimics only the targeted neurochemical aspect (i.e., cholinergic deficit) of cognitive impairment.

Previously, different kinds of pharmacological agents were tested in combination with memantine in preclinical behavioral models of cognitive impairment. Most frequently, memantine was combined with FDA-approved AChEI compounds, such as donepezil and galantamine. The combination of donepezil and memantine exerted no superior effect over monotreatments in preclinical animal experiments (Wise and Lichtman, 2007; Woodruff-Pak et al., 2007; Ihalainen et al., 2011). However, only one dose of donepezil was typically applied in these studies raising the possibility that negative results arose from the combinations of inappropriate doses. Moreover, meta-analyses of clinical studies in humans concluded that adjunctive memantine treatment showed no or only small benefits over AChEI monotherapies (Farrimond et al., 2012; Tsoi et al., 2016). Conversely, galantamine, an AChEI also acting as a positive allosteric modulator (PAM) on alpha7 nAChRs, influenced the efficacy of memantine in some preclinical animal models. Busquet et al. (2012) found a superior combinational effect of 0.1 mg/kg galantamine and 0.5 mg/kg memantine over monotreatments in spontaneous alternation and novel object recognition (NOR) paradigms of mice, using the scopolamine-induced amnesia model. Nikiforuk et al. (2016) reported similar results in rats using attentional set shifting and NOR tests, and further demonstrated that the interaction of galantamine (0.3–1.0 mg/kg) and memantine (1 mg/kg) in their cognitive effect was dependent on alpha7 nAChRs. Interestingly, Schneider et al. (2013) found that the addition of memantine (0.5 mg/kg) counteracted the cognitive enhancer effect of an effective dose of galantamine (0.3 mg/kg) in a delayed matching-to-sample (DMTS) working memory task in rhesus monkeys. This showed an antagonistic interaction similar to the one we observed in rats in the present study when PHA-543613 was co-administered with an effective dose of memantine.

Interactions between galantamine and memantine in combined treatments were mainly explained by the alpha7 nAChR PAM activity of galantamine, while it was also demonstrated that other alpha7 PAMs can also enhance the efficacy of memantine (Nikiforuk et al., 2016). Since the mechanisms of alpha7 nAChR activation by PAMs and agonists are substantially different (Lendvai et al., 2013), our present results may extend earlier findings by revealing that a selective agonist of the alpha7 nAChR also shows different types of interactions with memantine (enhancement, but perhaps also antagonism) affecting its efficacy in ameliorating cognitive impairment.

Pharmacological interactions between memantine and alpha7 nAChR ligands are not surprising, since memantine also acts as an antagonist for alpha7 nAChRs. Moreover, the affinity of memantine for alpha7 nAChRs was found to be higher than its affinity for NMDARs in the mouse brain *in vitro* (Aracava et al., 2005). Accordingly, as it has been suggested, the action of memantine on alpha7 nAChRs might also contribute to its cognitive effects (Banerjee et al., 2005), especially in low doses. However, it has not been clarified yet whether the affinity of memantine to alpha7 nAChR is beneficial or disadvantageous regarding its cognitive enhancer efficacy.

Finally, we propose possible mechanistic explanations for the observed interactive effects of memantine and PHA-543613. One possible explanation involves the effects of memantine and PHA-543613 on glutamatergic signaling. According to the classic interpretation of the cognitive enhancer effects of memantine, it is suggested that memantine weakly antagonizes NMDARs and blocks their pathologic overactivation at extrasynaptic sites, thus, increasing the signal-to-noise ratio of glutamatergic transmission (Xia et al., 2010; Collingridge et al., 2013). Furthermore, activation of alpha7 nAChRs on presynaptic sites of glutamatergic terminals increases glutamate release (Marchi et al., 2002; Gomez-Varela and Berg, 2013), and facilitates NMDA-dependent glutamatergic responses of hippocampal pyramidal neurons (Bali et al., 2017). It is hypothesized that the blockage of pathologic NMDAR activation and the concurrent cholinergic facilitation of synaptic glutamate release may even synergistically improve the physiological glutamatergic signaling.

Another explanation for the interaction between memantine and PHA-543613 involves alpha7 nAChRs as the same target of both compounds. Taking into account that memantine is also an antagonist of the alpha7 nAChR while PHA-543613 is an agonist of the same receptor, their additive effects might be explained by the agonist-induced desensitization effect (Quick and Lester, 2002). In their commentary, Banerjee et al. (2005) also refer to similar effects of alpha7 nAChR agonists and antagonists, and imply that antagonism or desensitization of the alpha7 nAChR may result in beneficial physiological and cognitive effects. According to this hypothesis, subeffective doses of memantine may not inactivate a sufficient number of alpha7 nAChRs to exert behavioral effects. Here, desensitization of further alpha7 nAChRs by PHA-543613 may have potentiated memantine-induced effects by increasing the number of inactivated receptors to exceed the threshold for cognitive effects.

The present results do not allow us to choose one or the other from the above discussed explanations. However, the additive relationship between memantine and PHA-543613 in the present study, and the finding that the maximum efficacy of memantine was not further increased by the addition of PHA-543613, together may suggest that the cognitive enhancer effects of the two compounds arose from their possible action on the same receptor target. As such, the common target of memantine and PHA-543613 may most likely be the alpha7 nAChR. Thus, our results further support the presumption that alpha7 nAChRs may play an important role in the cognitive enhancer effects of memantine. However, as a limitation, the present results do not provide direct evidence on the receptor-level interaction of memantine and PHA-543613.

## Conclusion

Here, we firstly reported the additive interaction between a selective alpha7 nAChR agonist and memantine in their effects on cognition in rats. Ineffective memantine treatments were successfully improved by the addition of PHA-543613, although the co-administration of the alpha7 nAChR agonist and memantine did not exceed the maximum efficacy of memantine monotreatments. Thus, the preclinical relevance of our results is that applied doses of memantine might be decreased by addition of low doses of alpha7 nAChR agonists, while also providing the beneficial cognitive enhancer effects. Furthermore, the addition of alpha7 nAChR ligands to memantine treatment may extend the therapeutic dose range of memantine by providing stable efficacy in a wider dose range. As a possible explanation of our results we hypothesize that the cognitive enhancer effects of the two compounds and their co-administration treatment may originate from their effect on the alpha7 nAChR. However, this presumption requires further investigation on the cellular level.

## Data Availability Statement

The experimental data are freely available on Mendeley Data (doi: 10.17632/szzm8d5chy.1).

## Author Contributions

ZB and IH designed the study. ZB, NB, ST, RC, LN, and MT performed the experiments and processed experimental data. ZB and NB performed the statistical analyses. ZB, NB, ST and IH wrote the first draft of the manuscript. All authors have approved the final manuscript.

## Conflict of Interest Statement

ZB, LN, and IH received occasional research funding from Gedeon Richter Pharmaceutical Plc. (Budapest, Hungary) for projects that are not related to the research reported in the manuscript. The remaining authors declare that the research was conducted in the absence of any commercial or financial relationships that could be construed as a potential conflict of interest.

## References

[B1] Aracava Y. Pereira E. F. R. Maelicke A. Albuquerque E. X. (2005). Memantine blocks alpha7^∗^ nicotinic acetylcholine receptors more potently than N-methyl-D-aspartate receptors in rat hippocampal neurons. *J. Pharmacol. Exp. Ther.* 312 1195–1205. 10.1124/jpet.104.077172 15522999

[B2] Bali Z. K. Bruszt N. Tadepalli S. A. Csurgyók R. Nagy L. V. Tompa M. (2018). Data for Cognitive enhancer effects of low memantine doses are facilitated by an alpha7 nicotinic acetylcholine receptor agonist in scopolamine-induced amnesia in rats. *Mendeley Data*. 10.17632/szzm8d5chy.1 PMC6371842 30804787

[B3] Bali Z. K. Inkeller J. Csurgyók R. Bruszt N. Horváth H. Hernádi I. (2015). Differential effects of α7 nicotinic receptor agonist PHA-543613 on spatial memory performance of rats in two distinct pharmacological dementia models. *Behav. Brain Res.* 278 404–410. 10.1016/j.bbr.2014.10.030 25447295

[B4] Bali Z. K. Nagy L. V. Hernádi I. (2017). Alpha7 Nicotinic Acetylcholine Receptors Play a Predominant Role in the Cholinergic Potentiation of N-Methyl-D-Aspartate Evoked Firing Responses of Hippocampal CA1 Pyramidal Cells. *Front. Cell. Neurosci.* 11:271. 10.3389/fncel.2017.00271 28928637 PMC5591832

[B5] Banerjee P. Samoriski G. Gupta S. (2005). Comments on “Memantine blocks alpha7^∗^ nicotinic acetylcholine receptors more potently than N-methyl-D-aspartate receptors in rat hippocampal neurons”. *J. Pharmacol. Exp. Ther.* 313 928–929. 10.1124/jpet.104.081976 15831445

[B6] Barber T. A. Meyers R. A. McGettigan B. F. (2010). Memantine improves memory for taste-avoidance learning in day-old chicks exposed to isolation stress. *Pharmacol. Biochem. Behav.* 95 203–208. 10.1016/j.pbb.2010.01.006 20100505

[B7] Busquet P. Capurro V. Cavalli A. Piomelli D. Reggiani A. Bertorelli R. (2012). Synergistic effects of galantamine and memantine in attenuating scopolamine-induced amnesia in mice. *J. Pharmacol. Sci.* 120 305–309. 10.1254/jphs.12166SC 23149577

[B8] Collingridge G. L. Volianskis A. Bannister N. France G. Hanna L. Mercier M. (2013). The NMDA receptor as a target for cognitive enhancement. *Neuropharmacology* 64 13–26. 10.1016/j.neuropharm.2012.06.051 22796429 PMC4696548

[B9] Dani J. A. Bertrand D. (2007). Nicotinic acetylcholine receptors and nicotinic cholinergic mechanisms of the central nervous system. *Annu. Rev. Pharmacol. Toxicol.* 47 699–729. 10.1146/annurev.pharmtox.47.120505.105214 17009926

[B10] Danysz W. Parsons C. G. G. (2012). Alzheimer’s disease, β-amyloid, glutamate, NMDA receptors and memantine–searching for the connections. *Br. J. Pharmacol.* 167 324–352. 10.1111/j.1476-5381.2012.02057.x 22646481 PMC3481041

[B11] Deacon R. M. J. Rawlins J. N. P. (2006). T-maze alternation in the rodent. *Nat. Protoc.* 1 7–12. 10.1038/nprot.2006.2 17406205

[B12] Deardorff W. J. Grossberg G. T. (2016). A fixed-dose combination of memantine extended-release and donepezil in the treatment of moderate-to-severe alzheimer’s disease. *Drug Des. Dev. Ther.* 10 3267–3279. 10.2147/DDDT.S86463 27757016 PMC5055113

[B13] Farrimond L. E. Roberts E. McShane R. (2012). Memantine and cholinesterase inhibitor combination therapy for alzheimer’s disease: a systematic review. *BMJ Open* 2 e000917–e000917. 10.1136/bmjopen-2012-000917 22689908 PMC3378937

[B14] Ferri C. P. Prince M. Brayne C. Brodaty H. Fratiglioni L. Ganguli M. (2005). Global prevalence of dementia: a delphi consensus study. *Lancet* 366 2112–2117. 10.1016/S0140-6736(05)67889-0 16360788 PMC2850264

[B15] Gomez-Varela D. Berg D. K. (2013). Lateral mobility of presynaptic α7-containing nicotinic receptors and its relevance for glutamate release. *J. Neurosci.* 33 17062–17071. 10.1523/JNEUROSCI.1482-13.2013 24155310 PMC3807030

[B16] Gould R. W. Garg P. K. Garg S. Nader M. A. (2013). Effects of nicotinic acetylcholine receptor agonists on cognition in rhesus monkeys with a chronic cocaine self-administration history. *Neuropharmacology* 64 479–488. 10.1016/j.neuropharm.2012.08.004 22921923 PMC3586788

[B17] Harada K. Nakato K. Yarimizu J. Yamazaki M. Morita M. Takahashi S. (2012). A novel glycine transporter-1 (GlyT1) inhibitor, ASP2535 (4-[3-isopropyl-5-(6-phenyl-3-pyridyl)-4H-1,2,4-triazol-4-yl]-2,1,3-benzoxadiazole), improves cognition in animal models of cognitive impairment in schizophrenia and Alzheimer’s disease. *Eur. J. Pharmacol.* 685 59–69. 10.1016/j.ejphar.2012.04.013 22542656

[B18] Holm S. (1979). A simple sequentially rejective multiple test procedure. *Scand. J. Stat.* 6 65–70. 10.2307/4615733

[B19] Ihalainen J. Sarajärvi T. Rasmusson D. Kemppainen S. Keski-Rahkonen P. Lehtonen M. (2011). Effects of memantine and donepezil on cortical and hippocampal acetylcholine levels and object recognition memory in rats. *Neuropharmacology* 61 891–899. 10.1016/j.neuropharm.2011.06.008 21704049

[B20] Kotermanski S. E. Johnson J. W. Thiels E. (2013). Comparison of behavioral effects of the NMDA receptor channel blockers memantine and ketamine in rats. *Pharmacol. Biochem. Behav.* 109 67–76. 10.1016/j.pbb.2013.05.005 23665480 PMC3723459

[B21] Lakens D. (2013). Calculating and reporting effect sizes to facilitate cumulative science: a practical primer for *t*-tests and ANOVAs. *Front. Psychol.* 4:863. 10.3389/fpsyg.2013.00863 24324449 PMC3840331

[B22] Lendvai B. Kassai F. Szájli A. Némethy Z. (2013). A7 nicotinic acetylcholine receptors and their role in cognition. *Brain Res. Bull.* 93 86–96. 10.1016/j.brainresbull.2012.11.003 23178154

[B23] Marchi M. Risso F. Viola C. Cavazzani P. Raiteri M. (2002). Direct evidence that release-stimulating alpha7^∗^ nicotinic cholinergic receptors are localized on human and rat brain glutamatergic axon terminals. *J. Neurochem.* 80 1071–1078. 10.1046/j.0022-3042.2002.00805.x 11953457

[B24] Nikiforuk A. Potasiewicz A. Kos T. Popik P. (2016). The combination of memantine and galantamine improves cognition in rats: the synergistic role of the α7 nicotinic acetylcholine and NMDA receptors. *Behav. Brain Res.* 313 214–218. 10.1016/j.bbr.2016.07.023 27435422

[B25] Parsons C. G. Danysz W. Quack G. (1999). Memantine is a clinically well tolerated N-methyl-D-aspartate (NMDA) receptor antagonist - A review of preclinical data. *Neuropharmacology* 38 735–767. 10.1016/S0028-3908(99)00019-2 10465680

[B26] Parsons C. G. Stöffler A. Danysz W. (2007). Memantine: a NMDA receptor antagonist that improves memory by restoration of homeostasis in the glutamatergic system–too little activation is bad, too much is even worse. *Neuropharmacology* 53 699–723. 10.1016/j.neuropharm.2007.07.013 17904591

[B27] Quick M. W. Lester R. A. J. (2002). Desensitization of neuronal nicotinic receptors. *J. Neurobiol.* 53 457–478. 10.1002/neu.10109 12436413

[B28] Rammes G. Danysz W. Parsons C. G. (2008). Pharmacodynamics of memantine: an update. *Curr. Neuropharmacol.* 6 55–78. 10.2174/157015908783769671 19305788 PMC2645549

[B29] Sadigh-Eteghad S. Talebi M. Mahmoudi J. Babri S. Shanehbandi D. (2015). Selective activation of α7 nicotinic acetylcholine receptor by PHA-543613 improves Aβ25–35-mediated cognitive deficits in mice. *Neuroscience* 298 81–93. 10.1016/j.neuroscience.2015.04.017 25881725

[B30] Schneider J. S. Pioli E. Y. Jianzhong Y. Li Q. Bezard E. (2013). Effects of memantine and galantamine on cognitive performance in aged rhesus macaques. *Neurobiol. Aging* 34 1126–1132. 10.1016/j.neurobiolaging.2012.10.020 23158762

[B31] Spowart-Manning L. van der Staay F. J. (2004). The T-maze continuous alternation task for assessing the effects of putative cognition enhancers in the mouse. *Behav. Brain Res.* 151 37–46. 10.1016/j.bbr.2003.08.004 15084419

[B32] Toyohara J. Hashimoto K. (2010). α7 Nicotinic receptor agonists: potential therapeutic drugs for treatment of cognitive impairments in schizophrenia and alzheimer’s disease. *Open Med. Chem. J.* 4 37–56. 10.2174/1874104501004010037 21249164 PMC3023065

[B33] Tsoi K. K. F. Chan J. Y. C. Leung N. W. Y. Hirai H. W. Wong S. Y. S. Kwok T. C. Y. (2016). Combination therapy showed limited superiority over monotherapy for alzheimer disease: a meta-analysis of 14 randomized trials. *J. Am. Med. Dir. Assoc.* 17 863.e1–8. 10.1016/j.jamda.2016.05.015 27349622

[B34] Wise L. E. Lichtman A. H. (2007). The uncompetitive N-methyl-d-aspartate (NMDA) receptor antagonist memantine prolongs spatial memory in a rat delayed radial-arm maze memory task. *Eur. J. Pharmacol.* 575 98–102. 10.1016/j.ejphar.2007.07.059 17850786 PMC2128866

[B35] Wishka D. G. Walker D. P. Yates K. M. Reitz S. C. Jia S. Myers J. K. (2006). Discovery of N-[(3R)-1-azabicyclo[2.2.2]oct-3-yl]furo[2,3-c]pyridine-5-carboxamide, an agonist of the alpha7 nicotinic acetylcholine receptor, for the potential treatment of cognitive deficits in schizophrenia: synthesis and structure–activity relationsh. *J. Med. Chem.* 49 4425–4436. 10.1021/jm0602413 16821801

[B36] Woodruff-Pak D. S. Tobia M. J. Jiao X. Beck K. D. Servatius R. J. (2007). Preclinical investigation of the functional effects of memantine and memantine combined with galantamine or donepezil. *Neuropsychopharmacology* 32 1284–1294. 10.1038/sj.npp.1301259 17119537

[B37] Xia P. Chen H. V. Zhang D. Lipton S. A. (2010). Memantine preferentially blocks extrasynaptic over synaptic NMDA receptor currents in hippocampal autapses. *J. Neurosci.* 30 11246–11250. 10.1523/JNEUROSCI.2488-10.2010 20720132 PMC2932667

[B38] Yang Y. Paspalas C. D. Jin L. E. Picciotto M. R. Arnsten A. F. T. Wang M. (2013). Nicotinic α7 receptors enhance NMDA cognitive circuits in dorsolateral prefrontal cortex. *Proc. Natl. Acad. Sci. U.S.A.* 110 12078–12083. 10.1073/pnas.1307849110 23818597 PMC3718126

